# Analysis of 114 Pedigrees of Renal Stone Patients: A Retrospective Review

**DOI:** 10.7759/cureus.13464

**Published:** 2021-02-21

**Authors:** Syed Raziuddin Biyabani, Jamsheer Talati, Daniya Umer, Zehra Kazmi, Hussamuddin Soomro, Rubab Mansoor

**Affiliations:** 1 Surgery, The Aga Khan University, Karachi, PAK; 2 Surgery, The Aga Khan University Hospital, Karachi, PAK; 3 Urology, The Aga Khan University, Karachi, PAK; 4 Pediatric Surgery, The Aga Khan University Hospital, Karachi, PAK

**Keywords:** renal stones, family history, stone recurrence, consanguinity

## Abstract

Background: Renal and ureteric stones (RS) can form due to genetic, metabolic, environmental, and diet-hydration related factors. Studies have shown that patients with family history (FH) of RS have higher likelihood of recurrence.

Materials and Methods: We conducted a retrospective cross-sectional study on 114 pedigrees to investigate the impact of FH on recurrence of RS and examine patterns of inheritance.

Results: Family history of renal stone disease was found in 42% of all patients. There was a significant increase of stone recurrence in RS patients with a positive FH (p=0.001). Seventy-one percent of patients with recurrent stones had at least one family member with RS. Interestingly, male penetrance was higher in RS recurrence, where a greater proportion of males had no FH of RS, indicating that there may be other factors involved as well.

Conclusion: Family history in RS patients should be continuously explored for the possible underlying genetic influence, whilst keeping in mind the dietary habits of the family.

## Introduction

Renal and ureteric stones (RS) form as a result of varied factors and influences. Genetic, metabolic, diet-hydration related factors, and pollution all play a role in the genesis of stones [1-5]. In a variable number of stone patients another family member is often diagnosed as having RS.

When a patient with RS has another member of the family member with the same condition, there is a likelihood that stones will most likely recur in that patient [6-7]. Other relatives may also develop stones, and possibly that too at a younger age [6]; the cause of stone in these cases may be genetic [8].

For each patient with a family history (FH), the possibility of an underlying genetic influence needs to be considered and explored, whilst keeping in mind the common eating and drinking habits of the family. Ljunghall, for example, noted that whilst stones formed significantly more commonly among the fathers and brothers of the proband cases than among the controls, there was also an increased frequency of stones in the (previously unrelated) wives, suggesting that environmental and/or dietary factors may be responsible [9]. When three generations are affected, however, a potential genetic cause should ideally be a part of the evaluation process. Ferraro recommends that all patients with a FH of RS should undergo investigation for excluding the possibility of a monogenic disease [1, 10]. If detected, the mode of inheritance for that disorder will be known and the physician will then be empowered to facilitate or provide genetic counseling. Patients can be informed about the possibility and chance of the disease in their offspring, the risks associated with future pregnancies, and the advisability of having children; in addition to strictly complying with ameliorating or preventive measures in children already born.

Thus, the finding of a history of renal stones in other members of the family is important as far reaching effects can result in streamlined management and control, including the pinpointing of a cause which might be amenable to specific targeted treatment, including liver and kidney transplants as for some forms of primary hyperoxaluria.

Recommendations [11] indicate the need for next generation sequencing or targeted analysis of suspected gene, according to the phenotype expressed, as gaged by the information from Fourier Transformation Infrared analysis of the stone, abnormalities in 24 hour urine or blood chemistry, or other phenotypic features.

The identification of patients with FH is advantageous to patients and their children. As the history of stones in other family members leads to more detailed investigation in even the first time stone former, the chances of pinpointing a cause and successfully reducing the individual’s odds of recurrence are enhanced.

The place of FH in the investigation of stone disease is firmly established. A pedigree would augment that usefulness by providing a visual representation of any apparent pattern of inheritance indicating whether the underlying genetic condition is autosomal/gender linked recessive or dominant.

Family history assessment in noncommunicable disorders such as RS is imperative as it alerts physicians to detailed investigation and optimized treatment regimens. If this leads to identification of a genetic disorder, this becomes important for the patient, and in addition can also be used in advising the relatives of the proposition that they are possibly also at risk.

In practice, maintaining a complete pedigree is seldom possible; but the larger the number of generations that can be captured the more valuable is this information. Our experience is that many patients do not know of all their relatives’ whereabouts, or their health status, especially that of second degree relatives. RS patients in Pakistan do not have knowledge of many of their relatives, at times cannot record their relatives in order of age, and have little idea of distant relatives who have often migrated to another town.

In the absence of electronic health records formats which include drawing of pedigree, we have commenced hand drawn recording of pedigrees. This retrospective report describes our analysis of 114 such pedigrees.

For this retrospective study, our data were already present in the system via clinical assessments. Within this data, we had noticed that a number of oxalate stone patients had a FH of stone. We therefore obtained institutional approval for a project to review patient pedigrees, with a hypothesis that more patients with oxalate stones would have another member of the family with RS, and that there would be a reduction in consanguineous unions.

## Materials and methods

After receiving institutional ethical board approval, this study was done through chart review and additionally, analysis of radiological and laboratory data, of patients who had attended the outpatient units (from January 1st, 2015 to June 30th, 2016) in the Section of Urology, Department of Surgery at Aga Khan University Hospital Karachi.

Data on renal stone patients coming to the outpatient clinics were observed via clinical notes assessment.

**Figure 1:**
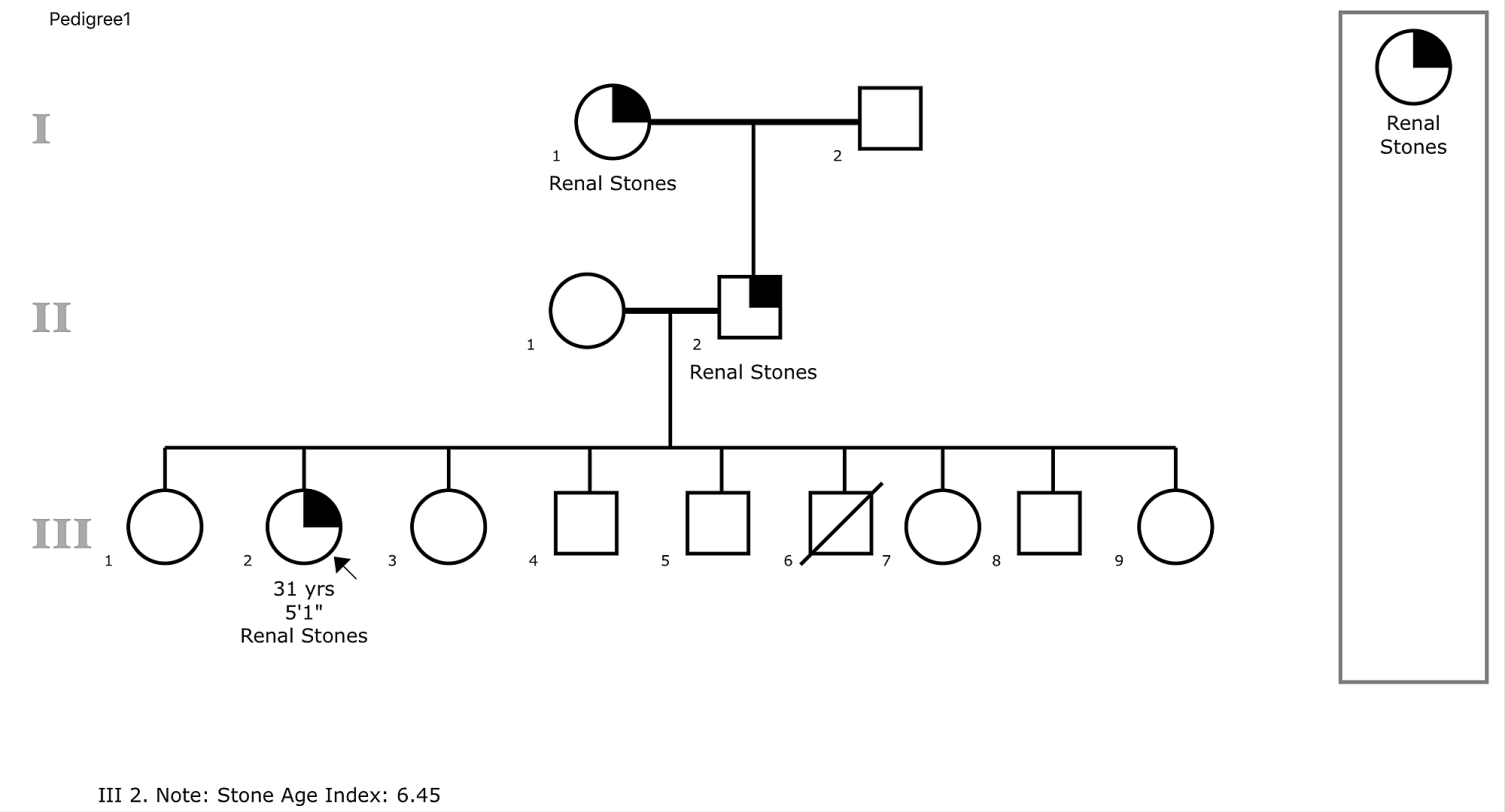
A pedigree drawn on Proband®. Circle: female; square: male; arrow denotes the patient

This retrospective cross-sectional analysis is an initial project conducted to review the pedigrees of 114 patients with renal stones attending one consultant’s clinic and in whom a FH was recorded as a hand drawn pedigree. The hand drawn pedigrees were first converted into a digital format on the Proband® application, The Children's Hospital of Philadelphia, Philadelphia, PA, USA (Figure 1).

Stone composition was noted when available. However, the most common surgical interventions for the treatment of RS (extracorporeal shock wave lithotripsy, intra-corporeal lithotripsy, and percutaneous nephron-lithotomy) all cause a fine dust which is difficult to collect for stone analysis. We therefore additionally analyzed the density of the stone in Hounsfield units (HU) where CT scans were done and available. As studies have shown that the attenuation coefficient of stones varies with stone composition, we considered RS with HU < 500 to be uric acid (UA) stones, and RS with HU ≥ 500 to be calcium stones [12].

Stone Age Index (SAI) of patients was computed using [13]:

$SAI=\frac{no of stone episodes}{Age}*100$

SAI ≥ 6 was considered high.

All the data were analyzed for relevant associations using Microsoft Excel and SPSS version 19. Continuous variables such as age, BMI, number of urolithiasis events, number of siblings with urolithiasis, SAI, HU were presented as mean ± SD. Categorical variables such as gender, consanguinity, stone recurrence, and family histories were recorded as frequencies and percentages. Means were compared using t-tests and categorical variables were compared using chi-square/Fischer Exact test. Nonparametric data sets were assessed using the Wilcoxon Rank Sum test. A p value of <0.05 was considered significant.

BMI was calculated from the recorded height and weight. A BMI ≥ 23 was considered high BMI, according to South Asian cut offs [14].

For the purpose of analysis, we divided the patients into two groups; those who had first or second degree relatives with RS (FH of RS) (Group 1), and those without (Group 2) any FH, as shown in Table 1.

**Table 1:** Comparing patients with family history of RS (Group 1), and patients with no family history of RS (Group 2). *This value was calculated using the Wilcoxon Rank sum test. **This value was calculated using the Fisher Exact test for proportions. RS, renal and ureteric stones; SAI, stone age index; BMI, body mass index

	Group 1 n=48	Group 2 n=66	p-value
SAI (Wilcoxon rank sum test)	6.67±1.07	4.16±0.52	0.0006*
SAI Males	5.28±0.73	4.28±0.66	0.0146
SAI Females	8.78±2.41	3.77±0.50	0.0492
BMI	27.78±0.91	27.18±0.79	0.6371
BMI Males	28.17±0.74	27.63±0.87	0.6748
BMI Females	25.67±1.61	28.27±2.77	0.4000
Recurrent stones	34 (70.83%)	25 (37.88%)	0.001**
Recurrent stones Males	20 (41.67%)	20 (30.30%)	0.01
Recurrent stones Females	14 (29.17%)	6 (9.09%)	0.04

## Results

The patient cohort

A total number of 114 RS (80 male and 34 female) pedigrees had been recorded. Female RS formers were 42.7 ± 2.98 years of age and males were 49.13 ± 1.8 years of age (p =0.027). There was no significant difference between the BMI of male and female renal stone formers (p=0.27). Similarly, no significant difference in BMI was found between recurrent RS formers and first time RS formers (p=0.93).

**Figure 2:**
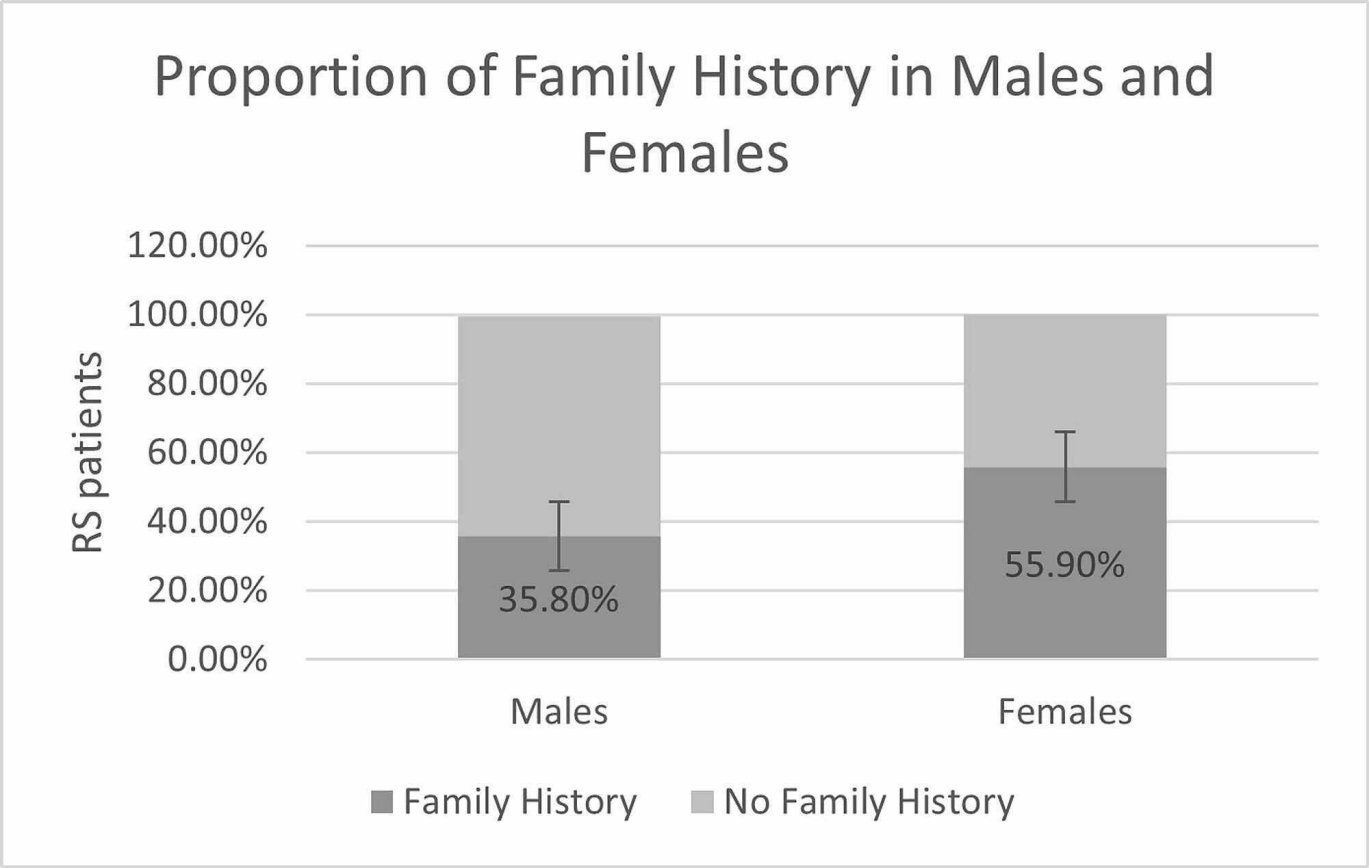
Only 35.8% of male RS formers had at least one family member affected with RS, whereas 55.9% of the female patients had family members suffering from RS. RS, renal and ureteric stones

Forty two percent of all RS patients had at least one member of their family suffering from kidney stones. Whilst male patients are greater in number, they make up a smaller proportion of patients with FH. Figure 2 shows this qualitative representation.

Male RS patients make up a lower proportion (29/80) of patients with positive FH, i.e. 35.8%, as compared to female patients, who have positive FH in 55.9% of patients. However, the difference is not significant (p=0.052).

Family history in RS formation

We found a significant difference between the SAI of patients in the two groups (p=0.0006), with a significantly higher SAI in patients in Group 1, in both males (p=0.0146) and females (p=0.0492).

However, there was no significant difference between BMI of both groups (p=0.6371). Recurrent stone formers were also found significantly higher in Group 1 (p=0.001). Interestingly, however, when stratified on the basis of gender, we found a significant increase in stone recurrence in male patients (p=0.01), and no significant difference in stone recurrence in female patients of both groups (p=0.08). Our results showed that 72.2% of RS patients with HU ≥ 500 were recurrent stone formers, and 47.4% of RS patients with HU < 500 were recurrent stone formers.

## Discussion

In order to examine time trends in RS patient’s FH, we compared the findings from this study with our earlier prospective study at the same institution in 1997 [15]. As mentioned in the results, we looked at clinically recorded data and found that 42.11% of the patients have a FH of RS as compared to 52% in the earlier cohort. There was no significant difference in first degree relatives who were affected between these cohorts (p=0.6715). However, there is a significant decrease in RS patients who have second degree relatives affected, i.e. maternal/paternal uncles/aunts, and cousins (p<0.0001). Moreover, there appears to be a significant reduction in the occurrence on consanguineous marriages as well (p=0.0017), with only 3.5% in this cohort, as compared to 16% in earlier cohort. In addition, we also saw that there is also a significant difference in patients with families that have three generations affected with RS (p<0.0001). Previously, 78.85% of the patients had RS in three generations of their families, whereas in this cohort, only 6.5% of patients have RS in three generations of their family.

One interesting trend that we observed in our current cohort was that there has been a significant reduction in consanguineous unions (p=0.0017). Out of 114 patients, only three patients were married to their cousins, and only one patient was a result of consanguineous union between parents. In previous studies, we saw that consanguineous unions attributed to 31%-62% of all marriages in Pakistan [16], and in our previous cohort (shown in Table 2), consanguineous marriages attributed to 16%. This difference may be due to the fact that workplaces and higher educational institutes have become places for opposite sexes to meet, and establish lifelong relationships. While in this cohort it appears that consanguinity has reduced, we cannot establish this as representative of Pakistan, since villages and farmlands may still have heavy rates of consanguinity.

**Table 2:** Comparative chart of results found in our previous cohort (1997) vs. current cohort (2019). RS, renal and ureteric stones

	2019 (n=114)	1997 (n=100)	p value
Males: Females	2.45: 1	2.8: 1	-
At least one member affected by RS disease	42.1% (n=48)	52% (n=52)	0.6715
Fathers affected	15 (31.25%)	21 (40.38%)	0.34
Mothers affected	10 (20.83%)	14 (26.92%)	0.48
Brothers affected	22 (45.83%)	16 (30.8%)	0.12
Sisters affected	9 (18.75%)	7 (13.46%)	0.47
Three generations affected	3 (6.25%)	41 (78.85%)	<0.0001
If father-son affected, what is the percent of all sons affected	40%	36.2%	-
If father-daughter affected, x% of all daughters affected	20%	26.31%	-
Maternal/paternal aunts/uncles and cousins affected	5 (10.42%)	25 (48.08%)	<0.0001
Grandparents affected	4 (8.3%)	8 (15.38%)	0.3
Parental consanguinity	4 (3.48%)	16 (16%)	0.0017

Also, with respect to our cohort, we may have to conduct an additional prospective study focusing on the presence of consanguinity amongst patients and its prevalence within our patients. Moreover, since we also saw that there was no significant difference between family members with RS in three generations and RS family members in a single generation, a greater sample size population may yield substantial significant differences.

Studies have also shown that RS patients with positive FH more often than less are likely to have calcium-based stones as compared to UA stones [10, 17]. McGeown saw a significant relationship between positive FH and high phosphate: creatinine clearance ratio [18]. Based on existing literature, we assumed that the CT scans which showed HU less than 500 are indicative of UA stones, and HU ≥ 500 are calcium stones [17]. While it appears that patients with UA stones, i.e. HU < 500, were less likely to form recurrent kidney stones in comparison to patients with calcium stones (HU ≥ 500), the differences were not significant and we cannot make a conclusion. However, if the sample size increases, we may see significant results. Studies have shown that most of the UA stones can be successfully controlled via dietary interventions [19-20]. Calcium stones (HU ≥ 500), on the other hand, may have an underlying genetic cause, in addition to a dietary cause, due to which recurrence may occur.

All of this information is interesting also in the fact that maybe stone clinics could pre-warn relatives of patients about their chance of forming a stone. A practical manner to do this would be the incorporation of nomograms that are based on pre-established trends and algorithms that can predict the occurrence and/or recurrence of nephrolithiasis.

Recording of FH is one factor that would increase the cost effectiveness of screening and development of algorithms for investigation. In our future research study plans, we are strategizing the idea of screening patients with three generations affected genetically for DNA mutations using whole exome sequencing. This has not yet been done in Pakistan for nephrolithiasis, and we believe we may find significant results that may help us develop effective and optimized treatment regimens.

Nevertheless, one cannot deny that RS recurrence may have another majorly influencing factor than the presence of FH. An interesting observation that was made was that there are less male patients with FH as compared to male patients without FH (Figure 2). Only 35.8% of male RS formers had the presence of FH while 64.2% had no history. Moreover, male recurrent RS formers were two times greater in proportion as compared to female recurrent RS formers (66.67%). Could lack of memory of recall be one of the reasons male patients appear to have a lower proportion of FH of RS? Social mobility allows many of the male patients to travel from all over the country to Karachi for treatment, however, many of these patients come without their families, especially wives, which may attribute to the lack of knowledge or memory of FH and past recurrences.

We also found that if father and son are affected, then 36.2% of all sons are affected, while only 26.3% of all daughters are affected if father-daughter are RS formers. One may attribute this fact to a common family diet, but it may also be an influence of the environment and pollution.

In Pakistan, women tend to have limited mobility outside their homes, whereas men travel for work outside homes [21]. Many men also migrate to Karachi for work, since it is an industrialized city, and end up eating low cost, possibly contaminated food, from the surroundings of their offices. Here, processed foods are increasingly available, and milk is preserved -- at times, contaminated with soya (a precursor of oxalate) [22]. Also, pollution may play a significant role in men forming RS more commonly than women. People inhale glyoxal (a precursor of oxalate) [23], as they traverse the streets of the city which is engulfed in petrol exhaust fumes, fully exposed as they commonly travel on motorcycles.

In both of the studies conducted at our institute, Talati [15], and the present study, we saw that the gender ratios were almost similar, i.e. 2.8:1 and 2.45:1, with males being greater in number. Once could also argue that since men excrete lower levels of citrate as compared to women, they are slightly more predisposed to form RS [24]. We can postulate that the major cause of renal stones is pollution or ingestion of contaminated/processed food, and inhalation of city’s toxic petroleum products in exhaust fumes, to which working fathers and husbands are exposed to a greater extent; and protected to a lesser extent because of lower citrate excretions [24].

## Conclusions

Migration to towns and industrial zones is an activity initiated mainly by the male members of a community, in search of better jobs, and an overall improved quality of life. The high index of consanguinity in Pakistan must be contributing to a higher pool of individuals with recessive genes. The simultaneous occurrence of heterozygous mutations and pollution could in today’s world spirally enhance the creation of plaques and concretions that result in stones.

In conclusion, in this study we found a positive FH in 42% of our renal stone patients; this is higher in females, leads to higher SAI and recurrence rates. The trend to consanguinity is decreasing as compared to our previous studies. Recording of a detailed FH through a pedigree, and then their visual translations made it easy to identify which relatives were affected with renal stones. In addition, we had the ability to add notes to these digital recordings, as per the requirements of the study. Digitizing pedigrees and recording them in routine clinical notes will help select patients with high risk of recurrence for appropriate counseling and referral for further genetic analysis.
